# SPIO-enhanced MRI for differentiating metastatic from reactive hyperplastic lymph nodes in breast cancer: diagnostic performance and association with VEGF-C expression

**DOI:** 10.3389/fmed.2026.1800084

**Published:** 2026-04-22

**Authors:** Tao Wu, Angela Wu, Hui Xiong, Jiaying Deng, Zhewen Zhang, Dan Gong, Jun Luo, Dan Wu

**Affiliations:** 1 Department of Cardiology, The Second Affiliated Hospital of Nanchang University, Nanchang, Jiangxi, China; 2 Department of Health Sciences, Queen’s University, Kingston, ON, Canada; 3 School of Medical Imaging, Nanchang Medical College, Nanchang, Jiangxi, China; 4 Department of Neonatology, The First Affiliated Hospital of Nanchang University, Nanchang, Jiangxi, China; 5 Department of Pathology, Nanchang People’s Hospital, Nanchang, Jiangxi, China; 6 First Department of Internal Medicine, Xiajiang County People’s Hospital, Xiajiang, Jiangxi, China

**Keywords:** breast neoplasms, lymphatic metastasis, magnetic resonance imaging, superparamagnetic iron oxide, vascular endothelial growth factor C

## Abstract

**Background:**

Accurate assessment of lymph node status is essential for staging and treatment planning in breast cancer. Conventional morphological imaging criteria have limited sensitivity and specificity. Superparamagnetic Iron Oxide (SPIO)-enhanced MRI offers a functional imaging alternative exploiting macrophage-mediated iron uptake. This study evaluated the diagnostic performance of SPIO-enhanced MRI in differentiating metastatic from reactive hyperplastic lymph nodes and explored the association between MRI signal characteristics and Vascular Endothelial Growth Factor C (VEGF-C) expression.

**Methods:**

Sixty female breast cancer patients (January–May 2024) underwent preoperative plain and SPIO-enhanced MRI (Resovist, 0.2 mL/kg, 12-h delay). Among 112 harvested lymph nodes, Hematoxylin and Eosin staining, Prussian Blue staining, and VEGF-C immunohistochemistry were performed. The Percentage of Signal Intensity Loss (PSIL) was calculated on T2*-weighted images. The primary diagnostic analysis was patient-level using mean PSIL with receiver operating characteristic analysis.

**Results:**

Of 112 lymph nodes from 60 patients, 47 were metastatic and 65 reactive hyperplastic. Reactive nodes showed marked signal attenuation (mean PSIL 64.7 ± 12.0%) compared with metastatic nodes (11.6 ± 7.8%; *p* < 0.001). Patient-level ROC analysis yielded an area under the curve of 0.84 (95% CI: 0.73–0.95). At a data-derived optimal cutoff of 42% (Youden index), sensitivity was 83%, specificity 85%, positive predictive value 80.0%, negative predictive value 87.2%, and overall diagnostic accuracy 84.0%. Histopathological Prussian Blue grading supported differential iron uptake patterns between groups. High VEGF-C expression (≥++) was more prevalent in metastatic than hyperplastic nodes (85.1% vs. 12.3%; *p* < 0.001). PSIL showed a moderate inverse correlation with VEGF-C grade (Spearman *r* = −0.67; *p* < 0.001).

**Conclusion:**

SPIO-enhanced MRI demonstrated favorable diagnostic performance for differentiating metastatic from reactive hyperplastic lymph nodes at the patient level. The imaging characteristics were associated with supportive histopathological findings and VEGF-C expression patterns. These findings warrant validation in larger, multicenter studies using currently available iron oxide agents.

## Introduction

Breast cancer remains the most prevalent malignancy among women globally, with the status of axillary and regional lymph nodes serving as a pivotal determinant for Tumor-Node-Metastasis (TNM) staging, prognosis, and adjuvant therapy planning (1). The clinical standard for assessing lymph node involvement relies on postoperative pathology or sentinel lymph node biopsy (SLNB). However, extensive lymph node dissection is invasive and associated with complications including lymphedema, nerve injury, and shoulder dysfunction (2, 3). A non-invasive imaging approach capable of accurately distinguishing metastatic lymph nodes from benign reactive hyperplasia would represent a meaningful clinical advance.

Conventional imaging modalities, including ultrasound, computed tomography (CT), and standard magnetic resonance imaging (MRI), primarily rely on morphological criteria such as lymph node size, shape, and margin characteristics (4, 29). However, lymph node size does not reliably predict malignancy: micrometastases may exist in normal-sized nodes, while inflammatory reactive hyperplasia can cause significant enlargement. This morphological overlap results in an overall diagnostic accuracy of only approximately 65%–70% for conventional assessment (5).

Superparamagnetic Iron Oxide (SPIO) nanoparticles are negative MRI contrast agents that target the reticuloendothelial system, particularly tissue macrophages (6, 17, 26). In benign or hyperplastic lymph nodes, resident macrophages phagocytose SPIO particles, producing signal attenuation on T2*-weighted sequences. In metastatic nodes, replacement of normal lymphoid tissue by tumor cells reduces macrophage density and SPIO uptake, resulting in preserved signal intensity (7, 23–25, 27). Prior studies have demonstrated the potential of iron oxide-enhanced MRI for lymph node staging across several cancer types, with a meta-analysis by Will et al. supporting the diagnostic precision of nanoparticle-enhanced MRI (8–10, 19, 28).

Vascular Endothelial Growth Factor C (VEGF-C) is a key mediator of lymphangiogenesis, acting on the lymphatic endothelial receptor VEGFR-3 to promote lymphatic vessel proliferation and dilation (11, 12, 18). VEGF-C overexpression has been associated with lymph node metastasis in breast cancer and several other malignancies (13, 14, 20). The relationship between SPIO-MRI signal patterns and molecular markers of lymphangiogenic activity such as VEGF-C has not been systematically investigated.

This study aimed to: (1) evaluate the diagnostic performance of SPIO-enhanced MRI in differentiating metastatic from reactive hyperplastic lymph nodes in breast cancer patients using quantitative signal analysis, and (2) explore the association between MRI signal characteristics and VEGF-C expression to provide biological context for the observed imaging patterns.

## Methods

### Ethics statement

This study was approved by the Ethics Committee of Nanchang People’s Hospital (Approval No. K-kt2026001) and conducted in accordance with the Declaration of Helsinki. Written informed consent was obtained from all participants prior to enrollment.

### Study design and population

This prospective, single-center diagnostic accuracy study enrolled 60 female patients with pathologically confirmed breast cancer admitted between January and May 2024. Inclusion criteria were: (1) breast cancer confirmed by core needle biopsy; (2) no prior chemotherapy, radiotherapy, or endocrine therapy; and (3) presence of evaluable cervical or axillary lymph nodes (short axis ≥ 5 mm) on baseline MRI. Exclusion criteria were: (1) contraindications to MRI; (2) known hypersensitivity to iron preparations; and (3) severe hepatic or renal dysfunction. Patients ranged in age from 28 to 68 years (median: 49 years). Each patient contributed between 1 and 4 lymph nodes for analysis (median: 2 nodes per patient), yielding a total of 112 evaluable lymph nodes.

### MRI acquisition protocol

All examinations were performed using a GE 3.0T superconducting MRI scanner with a dedicated breast coil. Patients were positioned supine. A pre-contrast scan was performed, including axial T1-weighted imaging (T1WI; TR 400–600 ms, TE 15–20 ms) and axial/coronal fat-suppressed T2-weighted imaging (T2WI-FS; TR 3,000–4,000 ms, TE 50–120 ms), with slice thickness 3 mm, gap 1 mm, and matrix 256 × 256. The SPIO contrast agent (Resovist; Bayer Schering Pharma; ferucarbotran) was administered intravenously at a dose of 0.2 mL/kg (approximately 0.56 mg Fe/kg), followed by a saline flush. A delayed post-contrast scan was performed 12 h after injection using identical T2WI-FS and T2*-weighted gradient-echo sequences (TR 300–500 ms, TE 15–25 ms, flip angle 20°).

### Image analysis

Two senior radiologists with over 5 years of experience, blinded to pathological results, independently analyzed all images. Morphological features (location, short-axis diameter, margin regularity, hilum preservation, internal signal homogeneity) were recorded. Signal intensity (SI) was measured by placing a region of interest (ROI) on the largest cross-section of each lymph node on T2*-weighted images, avoiding necrotic, cystic, and fatty areas. The Percentage of Signal Intensity Loss (PSIL) was calculated as: PSIL = (SI_pre − SI_post) / SI_pre × 100%.

Interobserver agreement for PSIL measurement was assessed using the intraclass correlation coefficient (ICC). Discrepancies in qualitative assessments were resolved by consensus. The “black-hole sign” was defined as complete or near-complete signal void within a lymph node on post-SPIO T2*-weighted imaging.

The optimal PSIL cutoff for distinguishing metastatic from non-metastatic lymph nodes was determined by maximizing the Youden index (J = sensitivity + specificity − 1) on the patient-level ROC curve. This was a data-derived threshold and was not prespecified from external literature.

### Histopathological examination

Lymph node dissection or biopsy was performed within 3 days of the MRI scan. Specimens were correlated with MRI findings based on anatomical region (axillary level or cervical station) and intraoperative surgical landmarks; strict spatial co-registration between imaging and pathological specimens was not performed. Specimens were fixed in 10% neutral formalin, paraffin-embedded, and serially sectioned at 4 μm. Hematoxylin and Eosin (H&E) staining was performed for diagnostic classification. Prussian Blue staining was performed on serial sections to detect iron particle deposition, with positive staining defined as blue cytoplasmic granules within macrophages. Prussian Blue staining intensity was graded semi-quantitatively as: negative (−), weak (+), moderate (++), or strong (+++).

### Immunohistochemistry

VEGF-C protein expression was detected using the streptavidin-peroxidase (SP) immunohistochemical method. Sections were deparaffinized, rehydrated, and subjected to heat-mediated antigen retrieval in citrate buffer (pH 6.0). Endogenous peroxidase was blocked with 3% hydrogen peroxide. Sections were incubated with mouse anti-human VEGF-C monoclonal antibody (dilution 1:100) overnight at 4 °C, followed by biotinylated secondary antibody and streptavidin-HRP conjugate. Visualization was performed with 3,3′-diaminobenzidine (DAB) chromogen.

VEGF-C expression was scored semi-quantitatively based on staining intensity (0 = none, 1 = weak, 2 = moderate, 3 = strong) and the percentage of positive tumor cells (0 = 0%, 1 = 1%–25%, 2 = 26%–50%, 3 = 51%–75%, 4 = 76%–100%). The product of intensity and percentage scores yielded a composite score ranging from 0 to 12. Expression was classified as: negative (−; score 0), weak (+; score 1–3), moderate (++; score 4–8), or strong (+++; score 9–12). High expression was defined as ≥++ (composite score ≥4), consistent with established conventions for semi-quantitative IHC scoring of angiogenic and lymphangiogenic growth factors (15, 16).

All histopathological and immunohistochemical evaluations were performed independently by two experienced pathologists blinded to MRI findings. Discrepancies were resolved by consensus review. Interobserver agreement for VEGF-C grading was assessed using weighted Cohen’s kappa.

### Statistical analysis

Data were analyzed using SPSS version 25.0. Normality of continuous variables was assessed using the Shapiro–Wilk test. Normally distributed continuous variables were expressed as mean ± standard deviation and compared using the independent-samples *t*-test; non-normally distributed variables were compared using the Mann–Whitney U test. Categorical data were analyzed using the chi-square test or Fisher’s exact test (when expected cell counts were <5).

The primary diagnostic analysis was conducted at the patient level. For each patient, mean PSIL was calculated by averaging PSIL across all sampled lymph nodes. Patients were classified as metastatic-positive if at least one lymph node was histopathologically confirmed as metastatic. Receiver operating characteristic (ROC) analysis was performed with mean PSIL as the test variable and patient-level metastatic status as the reference standard. Sensitivity, specificity, positive predictive value (PPV), and negative predictive value (NPV) were calculated at the optimal cutoff with 95% confidence intervals.

As a secondary analysis, node-level ROC analysis was performed treating each lymph node as an independent observation. To account for potential intra-patient clustering, a generalized estimating equation (GEE) with an exchangeable working correlation structure and logit link function was used to validate the node-level diagnostic performance.

Spearman rank correlation analysis was used to assess the association between PSIL and ordinal VEGF-C expression grade. All tests were two-sided; *p* < 0.05 was considered statistically significant.

## Results

### Patient and lymph node characteristics

A total of 112 lymph nodes from 60 patients were included in the analysis (median: 2 nodes per patient; range: 1–4). Histopathological examination classified 47 lymph nodes (42.0%) as metastatic and 65 (58.0%) as reactive hyperplastic. At the patient level, 28 patients (46.7%) had at least one metastatic lymph node and 32 (53.3%) had exclusively hyperplastic nodes.

### Pre-contrast morphological MRI findings

On pre-contrast T2-weighted imaging, metastatic lymph nodes had a slightly larger mean short-axis diameter compared with reactive hyperplastic nodes (1.28 ± 0.44 cm vs. 1.14 ± 0.35 cm; *p* < 0.05). Substantial size overlap was observed between groups, particularly in the 0.8–1.5 cm range. The overall diagnostic accuracy of conventional morphological criteria alone was limited (Table 1).

**Table 1:** Comparison of MRI and pathological characteristics between metastatic and reactive hyperplastic lymph nodes.

Characteristic	Metastatic group (*n* = 47)	Reactive hyperplastic group (*n* = 65)	*p* value
Short-axis diameter, cm	1.28 ± 0.44	1.14 ± 0.35	<0.05
Pre-contrast signal intensity	Predominantly hyperintense	Predominantly hyperintense	>0.05
PSIL, %	11.6 ± 7.8	64.7 ± 12.0	<0.001
Presence of black-hole sign, *n* (%)	5 (10.6)	39 (60.0)	<0.001
High Prussian Blue staining (≥++), *n* (%)	3 (6.4)	53 (81.5)	<0.001
VEGF-C IHC grade (− / + / ++ / +++)	0/7 / 15/25	37/20/7/1	—
High VEGF-C expression (≥++), *n* (%)	40 (85.1)	8 (12.3)	<0.001
Metastasis confirmed by H&E, *n* (%)	47 (100.0)	0 (0.0)	—

Values are presented as mean ± SD or n (%). p values were calculated using the independent-samples t test for continuous variables and the chi-square test or Fisher’s exact test for categorical variables, as appropriate. PSIL indicates percentage of signal intensity loss on post-SPIO T2*-weighted MRI. High Prussian Blue staining was defined as grade ≥++. High VEGF-C expression was defined as semi-quantitative IHC grade ≥++.

### SPIO-enhanced MRI: signal changes and diagnostic performance

**Figure 1:**
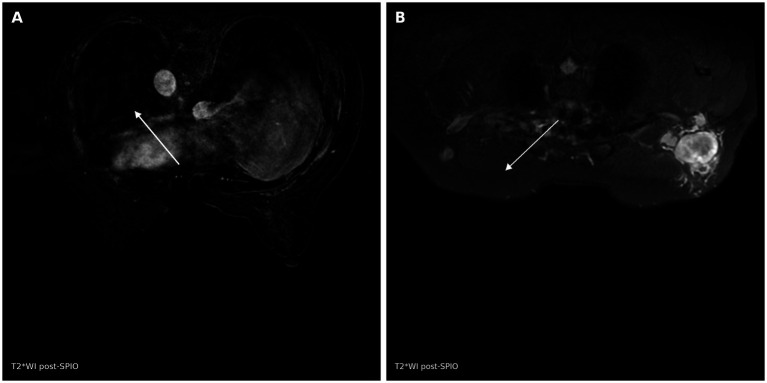
Representative SPIO-enhanced MRI appearances of lymph nodes. **(A)** Post-SPIO T2*-weighted image of a reactive hyperplastic axillary lymph node (arrow) demonstrating marked signal loss, consistent with macrophage-mediated iron uptake. **(B)** Post-SPIO T2*-weighted image of a metastatic axillary lymph node (arrow) showing preserved signal intensity, consistent with reduced iron uptake due to tumor cell replacement of normal lymphoid tissue. Field strength: 3.0 T; imaging performed 12 h after intravenous administration of SPIO (Resovist, 0.2 mL/kg).

Following SPIO administration, reactive hyperplastic lymph nodes demonstrated marked signal attenuation on T2*-weighted imaging (mean PSIL: 64.7% ± 12.0%), whereas metastatic lymph nodes exhibited minimal signal loss (mean PSIL: 11.6% ± 7.8%; *p* < 0.001) (Table 1). The black-hole sign was observed in 39 of 65 hyperplastic nodes (60.0%) compared with 5 of 47 metastatic nodes (10.6%; *p* < 0.001). Representative post-SPIO MRI images illustrating these signal differences are shown in Figure 1.

**Figure 2:**
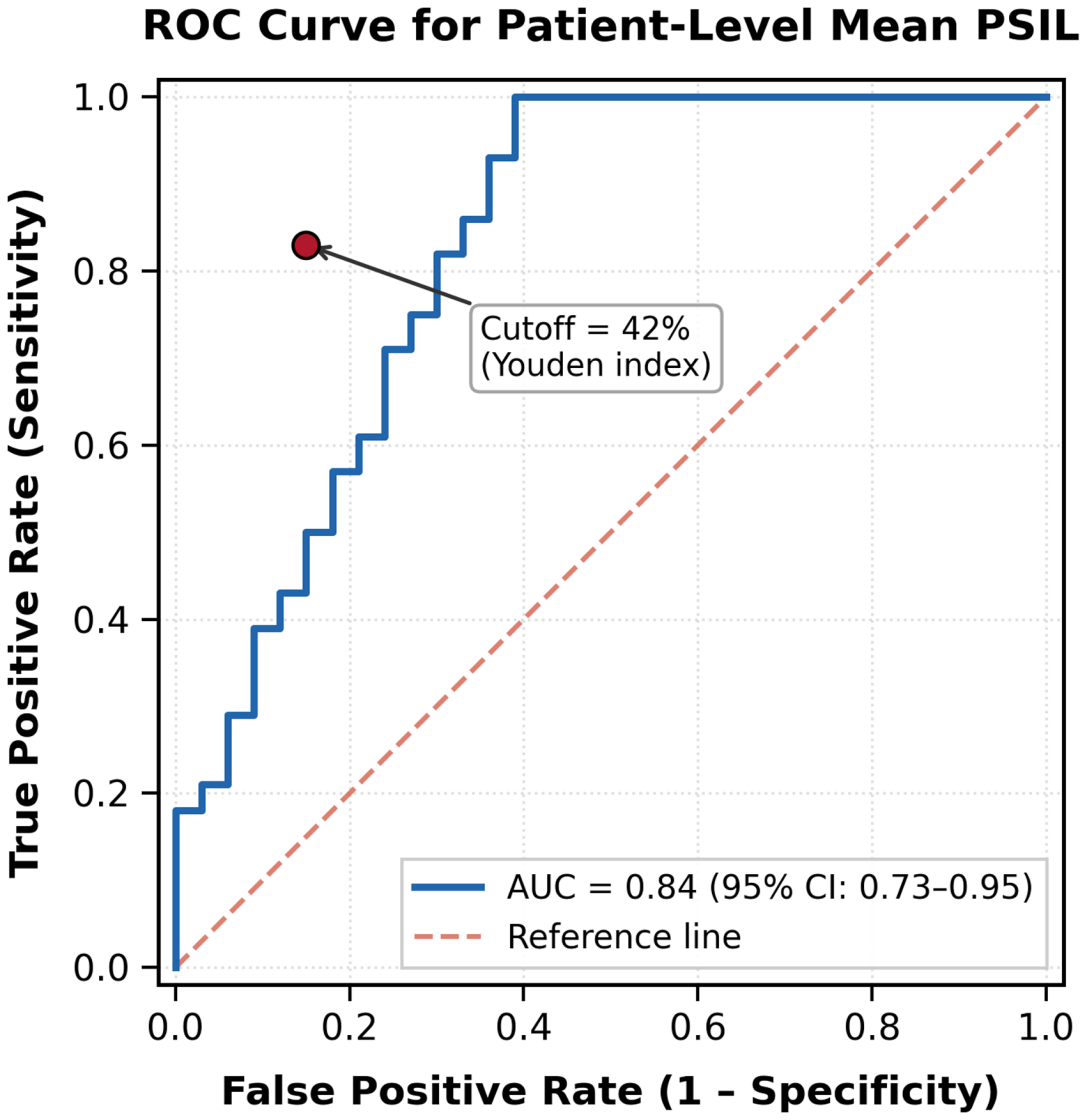
Receiver operating characteristic (ROC) curve for patient-level mean percentage of signal intensity loss (PSIL) in differentiating metastatic from non-metastatic lymph node status. The area under the curve (AUC) was 0.84 (95% CI: 0.73–0.95). The data-derived optimal cutoff of 42% (Youden index) achieved a sensitivity of 83% and specificity of 85%. The dashed diagonal line represents the reference line of no discrimination.

At the patient level, ROC analysis using mean PSIL yielded an area under the curve (AUC) of 0.84 (95% CI: 0.73–0.95) (Figure 2). The data-derived optimal cutoff of 42% (Youden index) achieved a sensitivity of 83%, specificity of 85%, positive predictive value of 80.0%, negative predictive value of 87.2%, and an overall diagnostic accuracy of 84.0% (Table 2). A clinically relevant gray zone was identified at PSIL values between 20% and 42%, within which 14 nodes (12.5%) from 10 patients fell; among these, 9 were metastatic and 5 hyperplastic, indicating that borderline cases may require additional clinical evaluation.

**Table 2:** Patient-level diagnostic performance of mean PSIL for identifying metastatic lymph node status.

Metric	Patient-level analysis
PSIL definition	Mean PSIL per patient
Optimal cutoff, %	42
Cutoff derivation	Youden index from patient-level ROC analysis
AUC	0.84
Sensitivity, %	83
Specificity, %	85
Positive predictive value (PPV), %	80.0
Negative predictive value (NPV), %	87.2
Overall diagnostic accuracy, %	84.0

Mean PSIL was calculated for each patient by averaging PSIL values across all sampled lymph nodes. Patients were classified as metastatic-positive if at least one lymph node was histopathologically confirmed as metastatic. The optimal cutoff of 42% was data-derived using the Youden index and has not been externally validated. PPV and NPV are prevalence-dependent and reflect the study population in the present cohort.

As a secondary analysis, node-level ROC yielded an AUC of 0.91 (95% CI: 0.86–0.96). GEE analysis with an exchangeable working correlation structure confirmed that intra-patient clustering did not materially alter the diagnostic estimates.

### Histopathological findings

**Figure 3:**
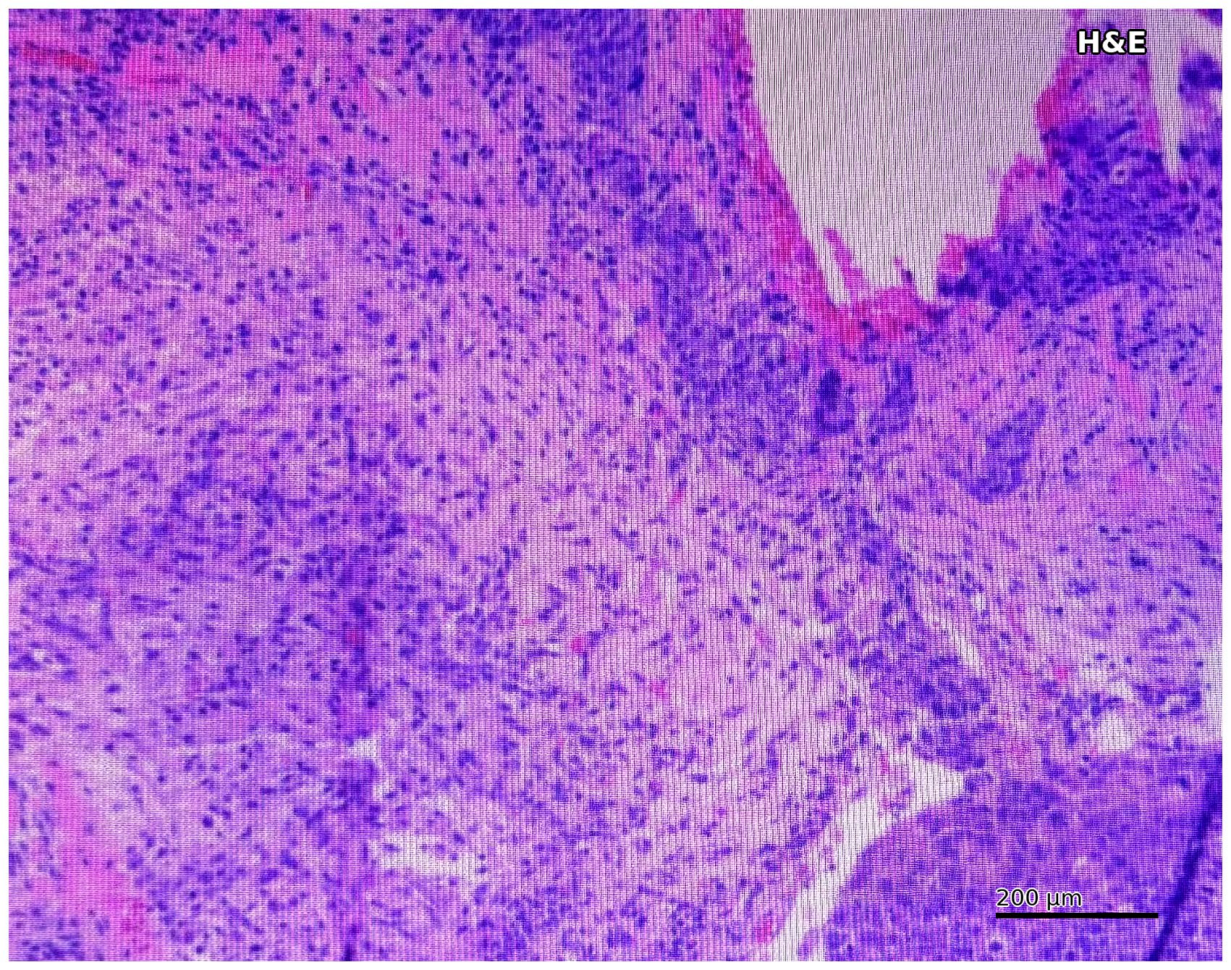
Hematoxylin and eosin (H&E)–stained section of a metastatic lymph node demonstrating tumor cell infiltration replacing normal nodal architecture.

Histopathological examination confirmed the classification of metastatic and reactive hyperplastic lymph nodes. Prussian Blue staining was performed on all specimens to assess iron particle deposition. Semi-quantitative grading demonstrated that high Prussian Blue staining (≥++) was significantly more frequent in reactive hyperplastic nodes than in metastatic nodes (81.5% [53/65] vs. 6.4% [3/47]; *p* < 0.001) (Table 1), consistent with the expected differential pattern of macrophage-mediated SPIO uptake. Representative H&E staining of a metastatic lymph node demonstrated tumor cell infiltration replacing normal nodal architecture (Figure 3). Direct spatial correspondence between individual histological sections and MRI voxels was not established.

### Association between VEGF-C expression and PSIL

**Figure 4:**
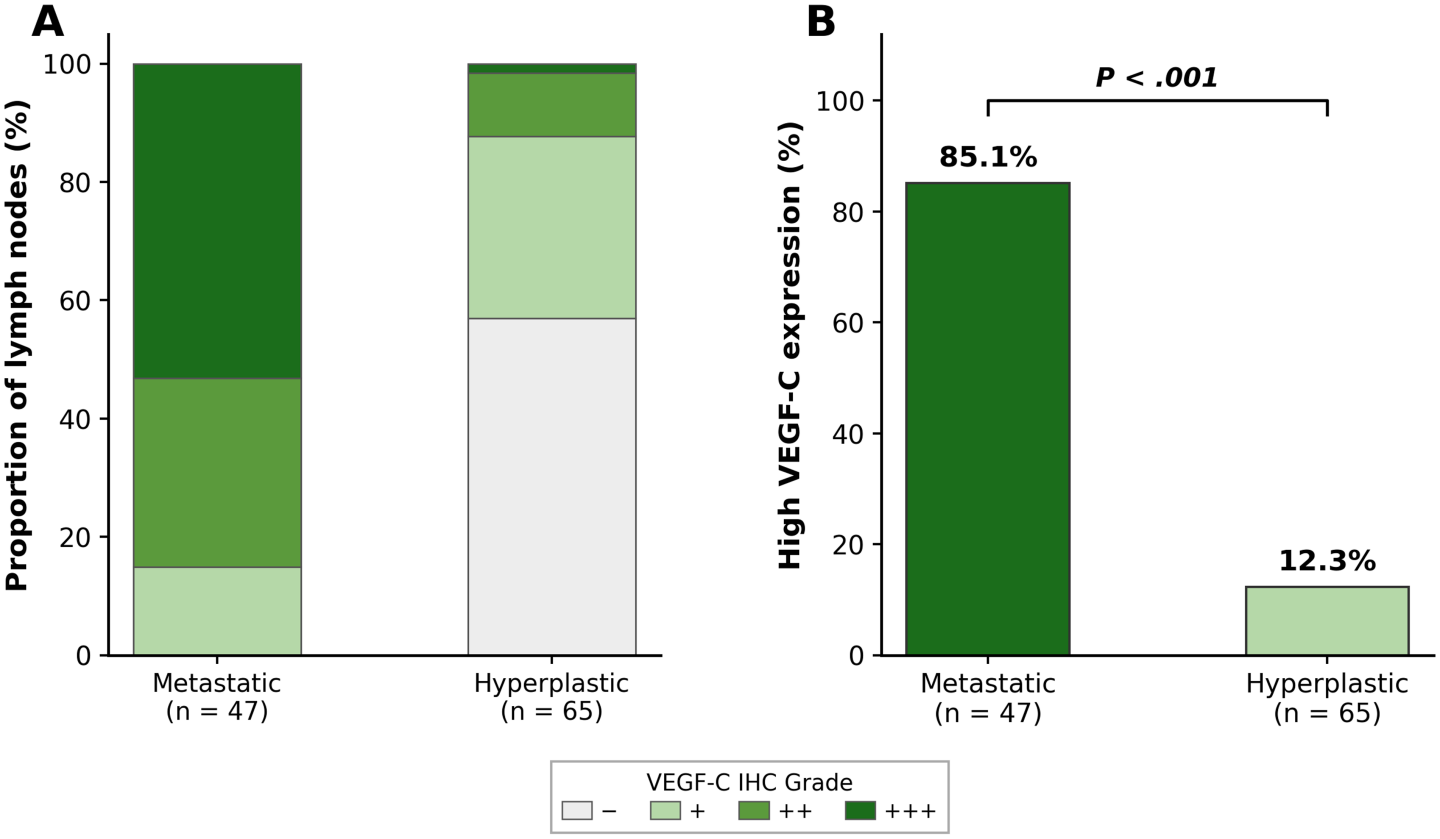
Distribution of VEGF-C expression across lymph node groups. **(A)** Stacked bar chart showing the proportion of lymph nodes in each VEGF-C immunohistochemical grade category (−, +, ++, +++) for metastatic (*n* = 47) and hyperplastic (*n* = 65) groups. **(B)** Comparison of the rate of high VEGF-C expression (≥++; composite score ≥4) between metastatic (85.1%) and hyperplastic (12.3%) groups (*P* < 0.001).

VEGF-C expression was assessed by immunohistochemistry across all lymph node specimens using a semi-quantitative composite scoring system (see Methods). The distribution of VEGF-C IHC grades differed markedly between the two groups (Figure 4A). High VEGF-C expression (≥++) was significantly more frequent in metastatic than hyperplastic nodes (85.1% [40/47] vs. 12.3% [8/65]; *p* < 0.001) (Table 1, Figure 4B).

PSIL values showed a moderate inverse correlation with VEGF-C expression grade (Spearman *r* = −0.67; 95% CI: −0.76 to −0.55; *p* < 0.001) (Supplementary Figure S1). Nodes with higher VEGF-C expression tended to have lower SPIO-induced signal attenuation, which may reflect a lymphangiogenic microenvironment in which both macrophage-mediated iron uptake and normal nodal architecture are disrupted.

### Safety

No SPIO-related adverse events were recorded during the study period. Adverse events were not prospectively monitored using a standardized toxicity grading system.

## Discussion

This prospective study evaluated the diagnostic performance of SPIO-enhanced MRI for differentiating metastatic from reactive hyperplastic lymph nodes in breast cancer and explored the association between quantitative MRI signal change and VEGF-C expression. Three principal findings emerged: (1) favorable patient-level diagnostic performance (AUC 0.84) using quantitative PSIL assessment; (2) supportive histopathological evidence of differential iron uptake confirmed by Prussian Blue grading; and (3) an inverse association between VEGF-C expression and PSIL, suggesting a biologically coherent imaging–pathological relationship.

The marked PSIL difference between reactive hyperplastic (64.7%) and metastatic (11.6%) lymph nodes reflects the differential macrophage content and phagocytic capacity of these nodal environments. In reactive nodes, abundant resident macrophages efficiently phagocytose SPIO particles, producing signal voids on T2*-weighted imaging. In metastatic nodes, replacement of normal lymphoid tissue by tumor cells reduces macrophage density and iron uptake, resulting in preserved signal intensity. These findings are consistent with prior iron oxide-enhanced MRI studies in multiple cancer types (7–10, 23–25, 31). The patient-level AUC of 0.84 represents an improvement over conventional morphological criteria and falls within the range reported in prior meta-analyses of nanoparticle-enhanced lymph node MRI (10).

Prussian Blue staining provided quantitative evidence of differential iron deposition between groups. The rate of high-grade staining (≥++) was markedly greater in the hyperplastic group (81.5%) than in the metastatic group (6.4%) (Table 1), supporting the biological basis of the observed MRI signal differences. H&E staining confirmed the histopathological classification of metastatic nodes (Figure 3). However, the available histological image evidence in this manuscript is limited to H&E demonstration; representative Prussian Blue micrographs could not be included due to archival material constraints. The quantitative Prussian Blue grading data reported in Table 1 nonetheless support a pattern consistent with macrophage-mediated SPIO uptake in reactive tissue and impaired uptake in tumor-infiltrated tissue. Additionally, dual immunohistochemical staining for iron and macrophage markers (e.g., CD68) was not performed, which would have provided more direct evidence of macrophage-specific iron uptake and represents an important direction for future investigation.

Several caveats apply to the diagnostic performance estimates. The 42% PSIL cutoff was internally derived via the Youden index and has not been externally validated; its generalizability to other populations and imaging protocols is uncertain. The node-level AUC (0.91) exceeded the patient-level AUC (0.84), likely reflecting the attenuation of discriminatory signal when averaging PSIL across multiple nodes per patient. PPV (80.0%) and NPV (87.2%) are prevalence-dependent and reflect the study sample composition (46.7% metastatic involvement at patient level).

The inverse association between VEGF-C expression and PSIL (Spearman *r* = −0.67) provides biological context for the imaging observations. VEGF-C is a key mediator of tumor lymphangiogenesis that has been consistently associated with lymph node metastasis in breast cancer (13, 14, 21). The finding that metastatic nodes with high VEGF-C expression exhibited the lowest PSIL values suggests that SPIO-enhanced MRI may capture functional imaging features associated with a lymphangiogenic nodal microenvironment. Importantly, this study was not designed to establish a causal relationship between VEGF-C expression and macrophage-mediated SPIO uptake. VEGF-C overexpression likely reflects a tumor-associated microenvironment in which normal nodal architecture, macrophage density, and iron uptake capacity are concomitantly disrupted, rather than VEGF-C directly regulating macrophage phagocytic function (22). The quantitative VEGF-C data (Figure 4) and the PSIL–VEGF-C correlation (Supplementary Figure S1) together provide supportive, though not mechanistically definitive, evidence for this interpretation.

### Limitations

This study has several limitations. First, this was a single-center study with 60 patients and 112 lymph nodes, limiting statistical precision. Multicenter validation with larger cohorts is essential.

Second, the SPIO protocol required a 12-h delay between contrast administration and post-contrast imaging, posing logistical challenges for routine clinical implementation including patient compliance and scheduling constraints. The present findings should be viewed as a proof-of-concept rather than a ready-to-deploy clinical protocol. Future studies should evaluate shorter-delay agents such as ferumoxytol.

Third, the SPIO agent used (Resovist/ferucarbotran) has been withdrawn from several markets (30). While the diagnostic principle is shared by other iron oxide agents, the specific PSIL thresholds may not transfer directly to other formulations.

Fourth, this study did not include direct comparisons with diffusion-weighted imaging (DWI) or dynamic contrast-enhanced MRI (DCE-MRI). Future comparative studies are warranted (32).

Fifth, MRI–pathology correlation was based on anatomical region and surgical landmarks rather than strict spatial co-registration. Both cervical and axillary nodes were included without site-specific stratification.

Sixth, VEGF-C was scored semi-quantitatively, which is inherently subject to interobserver variability. The ≥++ threshold is based on published convention rather than an independently validated biological cutpoint. Dual staining for iron and macrophage markers (e.g., CD68) was not performed.

Seventh, representative Prussian Blue and VEGF-C immunohistochemical micrographs could not be included in this manuscript due to archival tissue limitations. The histological evidence for differential iron uptake and VEGF-C expression is supported by quantitative grading data in Table 1. Accordingly, the interpretation of pathology–imaging concordance should be considered with appropriate caution.

Eighth, adverse events were not prospectively monitored using a standardized grading system.

Finally, the data-derived 42% PSIL cutoff requires external validation before clinical application.

## Conclusion

SPIO-enhanced MRI demonstrated favorable diagnostic performance for differentiating metastatic from reactive hyperplastic lymph nodes in this single-center prospective cohort. Quantitative assessment using PSIL at the patient level showed meaningful separation between groups, with supporting evidence from histopathological Prussian Blue grading data. VEGF-C expression differed significantly between metastatic and hyperplastic nodes and was inversely associated with PSIL, suggesting a biologically coherent, though not mechanistically confirmed, imaging–pathological relationship. These findings support the potential value of SPIO-enhanced MRI as a non-invasive adjunct for preoperative nodal assessment and warrant further validation in larger multicenter studies using currently available iron oxide agents.

## Data Availability

The raw data supporting the conclusions of this article will be made available by the authors, without undue reservation.

## Supplementary material

SUPPLEMENTARY FIGURE S1 Distribution of PSIL (%) across VEGF-C expression grades (−, +, ++, +++). A progressive decrease in median PSIL is observed with increasing VEGF-C grade (Spearman *r* = −0.67; *p* < 0.001). Boxes represent interquartile ranges; horizontal lines represent medians; whiskers extend to 1.5 × IQR; circles represent outliers. Number of lymph nodes per group: − (*n* = 37), + (*n* = 27), ++ (*n* = 22), +++ (*n* = 26).
