# Supplementary figures and images for: SPIO-enhanced MRI for differentiating metastatic from reactive hyperplastic lymph nodes in breast cancer: diagnostic performance and association with VEGF-C expression
Source: Front Med (Lausanne). 2026 Apr 22;13:1800084. doi: 10.3389/fmed.2026.1800084 (PMC13143616; doi:10.3389/fmed.2026.1800084)

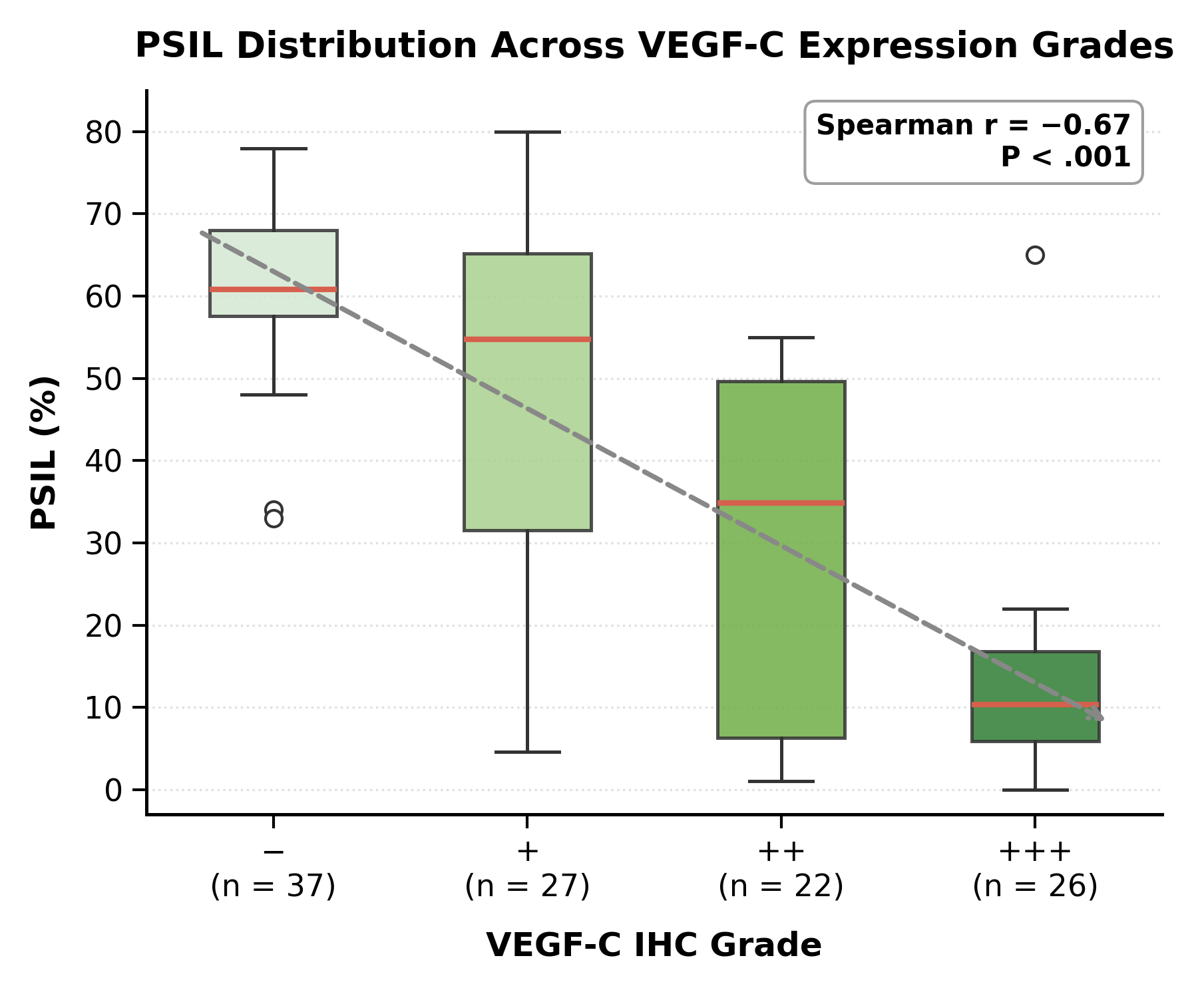

Supplement: SUPPLEMENTARY FIGURE S1 — Distribution of PSIL (%) across VEGF-C expression grades (−, +, ++, +++). A progressive decrease in median PSIL is observed with increasing VEGF-C grade (Spearman r = −0.67; p < 0.001). Boxes represent interquartile ranges; horizontal lines represent medians; whiskers extend to 1.5 × IQR; circles represent outliers. Number of lymph nodes per group: − (n = 37), + (n = 27), ++ (n = 22), +++ (n = 26). [file Image_1.tiff]
